# Urinary bisphenol A concentrations in girls from rural and urban Egypt: a pilot study

**DOI:** 10.1186/1476-069X-11-20

**Published:** 2012-04-02

**Authors:** Muna S Nahar, Amr S Soliman, Justin A Colacino, Antonia M Calafat, Kristen Battige, Ahmed Hablas, Ibrahim A Seifeldin, Dana C Dolinoy, Laura S Rozek

**Affiliations:** 1 Department of Environmental Health Sciences, School of Public Health, University of Michigan, Ann Arbor, MI, USA; 2 Department of Epidemiology, School of Public Health, University of Michigan, Ann Arbor, MI, USA; 3 Center for Global Health, University of Michigan, Ann Arbor, MI, USA; 4 National Center for Environmental Health, Centers for Disease Control and Prevention, Atlanta, GA, USA; 5 Tanta Cancer Center and the Gharbiah Cancer Society, Tanta, Egypt; 6 Department of Otolaryngology, University of Michigan, Ann Arbor, MI, USA

**Keywords:** Egypt, Urban, Rural, Bisphenol A

## Abstract

**Background:**

Exposure to endocrine active compounds, including bisphenol A (BPA), remains poorly characterized in developing countries despite the fact that behavioral practices related to westernization have the potential to influence exposure. BPA is a high production volume chemical that has been associated with metabolic dysfunction as well as behavioral and developmental effects in people, including children. In this pilot study, we evaluate BPA exposure and assess likely pathways of exposure among girls from urban and rural Egypt.

**Methods:**

We measured urinary concentrations of total (free plus conjugated) species of BPA in spot samples in urban (N = 30) and rural (N = 30) Egyptian girls, and compared these concentrations to preexisting data from age-matched American girls (N = 47) from the U.S. National Health and Nutrition Examination Survey (NHANES). We also collected anthropometric and questionnaire data regarding food storage behaviors to assess potential routes of exposure.

**Results:**

Urban and rural Egyptian girls exhibited similar concentrations of urinary total BPA, with median unadjusted values of 1.00 and 0.60 ng/mL, respectively. Concentrations of urinary BPA in this group of Egyptian girls (median unadjusted: 0.70 ng/mL) were significantly lower compared to age-matched American girls (median unadjusted: 2.60 ng/mL) according to NHANES 2009-2010 data. Reported storage of food in plastic containers was a significant predictor of increasing concentrations of urinary BPA.

**Conclusions:**

Despite the relatively low urinary BPA concentrations within this Egyptian cohort, the significant association between food storage behaviors and increasing urinary BPA concentration highlights the need to understand food and consumer product patterns that may be closing the gap between urban and rural lifestyles.

## Background

Bisphenol A (BPA) is a monomer used in epoxy resins and polycarbonate plastic production. BPA is one of the highest volume chemicals with worldwide annual production of more than 8 billion pounds [1]. BPA continues to play a fundamental role in the plastics industry, given that BPA-based plastic materials have a variety of desirable properties including transparency, high impact strength, malleability, and superior adhesive properties [2]. BPA-based materials have a broad range of applications and are found in many commonly used products such as water bottles, dental sealants, medical equipment, epoxy resin linings in food and beverage cans, and thermal paper. Exposure is nearly ubiquitous in the general U.S. population, with over 92% of individuals sampled in the 2003-2004 National Health and Nutrition Examination Survey (NHANES) having detectable BPA levels in their urine [3]. BPA has the ability to leach from consumer products such as baby bottles, water bottles, polyvinyl chloride (PVC) tubing, and plastic containers, creating many opportunities for exposure [4-8]. Diet is an important route of BPA exposure due to its leaching from food and beverage containers [9-11]. Other important routes of exposures include inhalation from aerosolized BPA and dermal uptake from contact with thermal paper, medical or dental supplies [12-15].

There has been growing interest in BPA exposure given its presence and use in both developed and developing countries, as well as the health implications associated with its ability to mimic endogenous estrogen. BPA has a lower binding affinity, interacting with the α and β estrogen receptors (ER) at 10^4 ^times lower potency compared to estradiol [16]. However, BPA has been shown to stimulate rapid cellular responses at concentrations much lower than anticipated, potentially due to high binding affinities for G-protein coupled receptor 30 (GPR30) and estrogen related receptor gamma (ERRγ) as seen in human studies [17,18]. BPA also antagonizes thyroid hormone and androgen function [16]. Both estrogenic and nonestrogenic effects from exposures to BPA *in vivo *and *in vitro *have been linked to changes in gene expression and cell proliferation in a variety of cells, tissues, and organs including the mammary glands and prostate [19,20]. Studies suggest that environmental exposure to BPA is associated with behavioral and reproductive abnormalities, as well as chronic diseases, especially when exposures occur during critical windows of development [21-25].

Exposure to endocrine disruptors varies within populations based on age, race, and sociodemographic factors [3,26]. Epidemiological studies conducted in Egypt also report geographical variance in disease incidence, highlighting the importance in evaluating the contribution of environmental exposures to these trends [27-29]. Developing countries, where there are major discrepancies in social and behavioral practices between urban and rural populations, offer a unique setting in which to study potential toxicant-disease interactions. Therefore, it is important to understand environmental exposure profiles in vulnerable populations, especially in developing countries where exposures remain largely uncharacterized. In this pilot study, we determined urinary concentrations of BPA in 60 premenstrual girls from urban and rural Egypt. BPA concentrations within the Egyptian cohort were compared to an age- and sex-matched United States population from NHANES 2009-2010. In addition, we investigated possible associations between urinary BPA concentrations and residential status, anthropometric measurements, and questionnaire data to assess potential sources of exposure.

## Methods

### Subject selection

In 2009, we recruited healthy females between the ages of 10-13 years living in either rural (N = 30) or urban (N = 30) areas of the Gharbiah province of Egypt, located 90 kilometers north of Cairo. Approximately 30% of Gharbiah's population resides in urban areas, with Tanta serving as the capital of all eight districts within the region. To determine rural versus urban status, participants were assigned a residence code based on their residential address that followed the Central Agency for Public Mobilization and Statistics (CAPMAS) national census coding of urban and rural areas [22]. To recruit urban subjects, we utilized a systematic random sampling of census records from Tanta. Rural subjects were recruited via systematic random sampling from two villages in two separate districts in the province. No refusals were encountered when selected subjects were approached in local primary schools to participate. All study participants were provided bus transportation to and from the Tanta Cancer Center and were not restricted from food and water access. Mothers of participants gave informed consent prior to sample collection. Approval from the Institutional Review Boards of the University of Michigan and the Gharbiah Cancer Society were obtained before starting the study. The involvement of the Centers for Disease Control and Prevention (CDC) laboratory was determined not to constitute engagement in human subject research.

### Urine sample collection and questionnaire

Participants provided one spot urine sample, completed an administered questionnaire in Arabic, and were measured for height, weight, waist, and hip circumference. Urine samples from individuals were collected between 12:00 and 4:00 PM in sterile polypropylene containers between July and October 2009. Nine mL of urine were mixed thoroughly, split into two aliquots, and shipped on dry ice to the National Center for Environmental Health (NCEH) at the CDC for analysis and the University of Michigan School Of Public Health for archiving. An interviewer administered a lifestyle and diet questionnaire in Arabic in order to assess potential routes of exposure. The questionnaire, entitled "Comparison of Xenoestrogen Levels Among Prepubertal Females in Urban and Rural Gharbiah, Egypt," contained questions addressing residential history, personal care product usage, family history of cancer, use of canned foods, and food preparation and storage behaviors.

### Urinary BPA measurements

The Division of Laboratory Sciences of NCEH, CDC determined the urinary concentrations of total (free plus conjugated forms) of BPA. Urine samples from each participant were subject to enzymatic hydrolysis of the conjugated (glucuronide and sulfate) BPA species and then automated cleanup. Total BPA was measured by on-line solid phase extraction coupled with isotope dilution-high performance liquid chromatography-atmospheric pressure chemical ionization-tandem mass spectrometry following a previously described analytical approach [30]. For quality assessment, in addition to the calibration standards, we analyzed two blanks, two low-concentration quality control (QC) materials (~2 ng/mL with individual measurements: 2.67 and 2.62 ng/mL), and two high-concentration QCs (~9 ng/mL with individual measurements: 9.04 and 9.47 ng/mL), along with the study samples. BPA was undetectable in the two blanks. Similar to measurements taken for NHANES, the limit of detection (LOD) at 0.4 ng/mL was determined using repeated measurements of low-level synthetic urine standards as previously described [30]. CDC personnel received coded specimens and did not have access to any personal, geographic, anthropometric, or questionnaire information from the study subjects.

### Statistical analysis

Urinary BPA concentrations below the LOD were assigned a value of 0.28, which was estimated by dividing the LOD by the square root of 2. Both creatinine, measured using a colorimetric assay, and specific gravity, measured by refractometry, were recorded for each participants in the field. Given the high inter-individual variability and subjective assessment of creatinine, we adjusted urinary BPA concentrations for specific gravity (SG), previously used as a reliable alterative to creatinine [31,32]. Briefly, specific gravity adjusted urinary BPA concentrations were calculated by the formula P_c _= P[(1.018 - 1)/(SG-1)], where P_c _is the specific gravity adjusted BPA concentration (ng/mL), P is the measured unadjusted urinary BPA concentration, 1.018 is the sample population median specific gravity value, and SG is the measured specific gravity for each urine sample. Samples from three girls had specific gravity levels below 1.005; these samples were excluded from the analyses because they were too dilute, resulting in a final sample size for BPA analysis of N = 57. Age, body mass index (BMI), the use of plastic containers for food storage, and the consumption of canned foods were obtained from the questionnaire and anthropometric measurements. Urban and rural status was assigned as previously described [22]. Univariate statistics, including mean, geometric mean (GM), geometric standard error, median, and range for demographic, anthropometric, and BPA measurements were calculated. Chi-square tests were used to compare food storage behaviors and a *t*-test was used to compare urinary BPA concentrations between rural and urban girls. Pearson correlation analyses were conducted to assess the association between log-transformed unadjusted urinary BPA concentrations and individual covariates. Both specific gravity adjusted and unadjusted urinary BPA concentrations were reported in the univariate analyses, while log-transformed unadjusted BPA concentrations were used in the bivariate and multiple regression models to correct for departures from normality of the regression residuals. Specific gravity was included as a covariate in the multivariate analysis for each regression model. Age, BMI, and specific gravity were included in the multivariate regression model as continuous biologically relevant variables, while canned food consumption, food storage in plastics, and residential status were included as categorical variables. Canned food use and food storage in plastics were modeled as yes versus no in response to the questions "Do you eat tinned or canned food products?" and "Do you store food in plastic bags, containers, and/or Tupperware?", respectively. Regression coefficients with *p *< 0.05 were considered statistically significant and β values were interpreted by calculating percent change [100(e^β -1)]. Univariate and multiple regression analyses were conducted using the UNIVARIATE and REG procedures in SAS software (version 9.2, SAS Institute, Cary, NC).

We also performed statistical analysis of urinary BPA concentrations from 47 age- and sex-matched NHANES 2009-2010 participants using the SURVEYMEANS procedures in SAS with relevant strata, clusters, domain, and weights taking into account the complex sampling design. NHANES analysis was restricted to girls between the age of 10 and 13, who submitted urine samples during the afternoon session to match the sampling of our Egyptian cohort. A two-sample *t*-test was used to compare geometric means of urinary BPA measurements from NHANES to urinary BPA concentrations in our Egyptian subjects.

## Results

Among the 60 girls recruited, three were excluded from analyses due to urine dilution (specific gravity < 1.005) resulting in a final sample size for analysis of N = 57. Exclusion of these subjects from our analysis did not significantly alter the overall distribution of anthropometric and urinary BPA measurements from this cohort. Anthropometric parameters including BMI, hip circumference, and waist circumference were similar between the 28 urban and 29 rural Egyptian girls in this study (Table 1). Participants' age ranged from 10-13 years with an average age of 11 years for both urban and rural groups. Overall, there were no significant differences in age and anthropometric measurements between the two groups. When we compared anthropometric measurements to age and sex matched NHANES participants, American girls exhibited higher BMI and waist circumference compared to Egyptian girls.

**Table 1:** Demographic and anthropometric measurements of urban vs. rural Egyptian^a ^girls and American^b ^girls

	Urban Egyptians (N = 28)		Rural Egyptians (N = 29)		NHANES (N = 47)	
	Mean, Median	Min, Max	Mean, Median	Min, Max	Mean, Median	Min, Max
Age (years)	11.4, 11.4	10.1, 13.2	11.6, 11.6	10.1, 13.6	11.3, 11.0	10.0, 13.0
Body Mass Index (kg/m^2^)	20.2, 18.6	13.8, 30.2	19.3, 19.1	13.7, 31.2	22.1, 21.3	14.5, 34.2
Hip Circumference (cm)	62.0, 60.6	35.5, 92.0	59.9, 59.3	48.5, 80.0	N/A, N/A	N/A, N/A
Waist Circumference (cm)	42.9, 42.0	31.0, 62.0	40.4, 40.7	33.0, 56.3	76.6, 74.0	55.2, 111

^a^Demographic and anthropometric measurements were similar before and after exclusion of 3 girls who provided diluted urine specimens (specific gravity < 1.005; recruited N = 60, analyzed N = 57)

^b^Girls age 10-13 years from NHANES 2009-2010

Bivariate analyses of food storage behaviors indicate significantly different reports of canned food consumption but similar reports of food storage in plastics between urban and rural girls (*p*-value = 0.001 and 0.248, respectively). Approximately 25% of girls residing in rural areas compared to 68% of those residing in urban areas reported consuming canned foods, while 69% of rural girls and 82% of urban girls reported storing food in plastic containers (Table 2). In general, 18% of rural girls and 61% of urban girls reported both canned food consumption and food storage in plastics within this population. Specific gravity was significantly lower among urban girls compared to rural girls (*p *= 0.035; data not shown).

**Table 2:** Percent of urban vs. rural Egyptian girls who responded "Yes" to food storage behaviors

	Total (N = 57)	Urban (N = 28)	Rural (N = 29)	*p*-value^b^
Canned Food Use^a^	46%	68%	25%	0.001*
Food Storage in Plastic	75%	82%	69%	0.248
Canned Food Use + Food Storage in Plastics	39%	61%	18%	0.001*

^a^One non-respondent (rural N = 28, total N = 56)

^b^Two-sided significance calculated from Pearson chi-squared

*Significance at *p*-value < 0.05

The majority of girls (90% rural and 68% urban) had detectable urinary BPA concentrations. Among the 57 study participants, both SG-adjusted and unadjusted urinary BPA measurements exhibited a right skewed non-normal distribution. Rural Egyptian girls had a median unadjusted urinary BPA concentration of 0.60 ng/mL (range = <LOD-12.0 ng/mL, GM = 0.89), while urban Egyptian girls had a median unadjusted urinary BPA concentration of 1.00 ng/mL (range = <LOD-6.2 ng/mL, GM = 0.79, *p *= 0.478, Table 3). After adjustment for specific gravity, median urinary BPA concentrations slightly increased to 1.08 ng/mL in rural girls and 1.02 ng/mL in urban girls (*p *= 0.578, Table 3). BPA urinary concentrations from an age- and sex-matched subset (N = 47) from NHANES 2009-2010 were significantly higher than Egyptian (N = 57) BPA measurements (GM: 1.96 ng/mL in NHANES, GM = 0.84 ng/mL in Egypt, *p *< 0.01, Table 3).

**Table 3:** Total urinary unadjusted and adjusted BPA concentrations (ng/mL) in Egyptian girls compared to American^a ^girls

BPA	N	% Above LOD	Mean	Median	Min	Max	Geom.Mean (GSE^b^)
Unadjusted							
All	57	79	1.34	0.70	<LOD	12.0	0.84 (0.10)
Urban	28	68	1.24	1.00	<LOD	6.20	0.79 (0.14)
Rural	29	90	1.44	0.60	<LOD	12.0	0.89 (0.89)
NHANES^a^	47	98	2.83	2.60	<LOD	16.1	1.96 (0.23)*

Specific Gravity							
Adjusted							
All	57		1.75	1.02	<LOD	10.8	1.00 (0.14)
Urban	28		2.13	1.02	<LOD	10.8	1.14 (0.25)
Rural	29		1.39	1.08	<LOD	8.64	0.89 (0.16)

^a^Girls age 10-13 years from NHANES 2009-2010

^b^GSE = Geometric standard error; LOD = 0.4 ng/mL

* Geometric means significantly differed between NHANES and Egypt cohorts when tested using two sample *t*-test (*p *< 0.01)

No significant correlations were observed between unadjusted log-BPA urinary concentrations and individual covariates such as age, BMI, specific gravity, residential status, and canned food consumption (data not shown). However, a significant Pearsons correlation was detected between log-BPA concentrations and food storage in plastic containers (*p *= 0.041). Furthermore, multiple linear regression analysis was performed to assess the strength of association between BPA concentration and our primary predictor, residential status, after adjusting for well-known covariates. In the final model, we adjusted for urban/rural status, canned food consumption, food storage in plastic, specific gravity, age, and BMI (Table 4). The association between urinary BPA concentrations and residential status was not significant after adjusting for biological covariates (β = -0.073 and *p *= 0.783). Given the significant correlation between food storage in plastic containers and BPA in the bivariate analysis, we assessed the relationship between BPA concentrations and food storage behaviors after adjusting for covariates. Of the two food storage behaviors analyzed (Table 4), storage in plastic containers was a significant predictor of increasing urinary BPA concentration after adjusting for covariates (β = 0.626 and *p *= 0.036). Adjustment of this model, especially by canned food use, improved the association between urinary BPA and food storage in plastic. Girls who reported storage of food in plastic containers had, on average, 87% higher urinary BPA concentrations compared to girls who did not store food in plastic containers after adjusting for biological covariates, canned food use, and residential status.

**Table 4:** Residential status, canned foods, and food storage in plastics as potential predictors of (log transformed) urinary BPA concentrations (ng/mL)

	β	95% CI	*p*-value	% change
Residential Status^a^	-0.072	[-0.577,0.434]	0.777	-6.95%
Residential Status^b^	-0.073	[-0.600,0.455]	0.783	-7.04%

Canned Food Use^a^	0.365	[-0.118,0.848]	0.136	44.05%
Canned Food Use^b^	0.394	[-0.108,0.895]	0.121	62.42%

Food Storage in Plastic^a^	0.552	[0.007,1.097]	0.047*	73.67%
Food Storage in Plastic^b^	0.546	[-0.012,1.105]	0.055	72.63%
Food Storage in Plastic^c^	0.626	[0.043,1.210]	0.036*	87.01%

^a^Adjusted for specific gravity

^b^Adjusted for specific gravity, BMI, and age

^c^Adjusted for all other covariates (specific gravity, BMI, age, residential status, plastic storage, and canned food)

*Significance at *p*-value < 0.05; predictors were run separately for models a, b, and c

## Discussion

Total urinary BPA, including free and conjugated species, was detected at low parts-per-billion levels in premenstrual Egyptian girls, 21% of whom exhibited concentrations below the limit of detection at 0.40 ng/mL. Overall, the Egyptian cohort showed significantly lower urinary BPA concentrations compared to age- and sex-matched American girls from NHANES 2009-2010. Similar ranges of BPA concentrations compared to this Egyptian cohort have been reported in several international cohorts. Recent studies report that the median concentration of urinary BPA in 48 Japanese women was 1.2 ng/mL [33], while the median BPA concentration of 60 pregnant Mexican women was 0.95 ng/mL [34] and 100 pregnant women in the Netherlands was 1.2 ng/mL [35]. Comparison of urinary BPA concentrations between countries may be confounded by the method of analysis, adjustment for urine dilution, timing of urine collection, as well as age, sex, and genetic differences [3,36,37]. However, differences can also be attributed to country specific lifestyle practices including food storage and consumer product use. Therefore, characterizing dietary and behavioral habits may provide insight into the potential exposure pathways and contribution of BPA to human health.

Previous studies have described heterogeneity in disease rates in Egypt based on urban/rural status. Women residing in urban Gharbiah Province of Egypt were found to have a 2-4 times higher incidence rate of estrogen receptor positive breast cancer compared to their rural counterparts [29]. Xenoestrogen exposure during critical windows of development has been proposed to be a likely explanation for increasing cancer incidences, especially in regards to breast cancer [22,27,38-41]. In this pilot study, however, we did not identify differential exposure to BPA, estimated from the urinary concentrations of BPA, based on residential status in premenstrual Egyptian girls. Both average unadjusted and SG-adjusted BPA concentrations were in the low parts-per-billion range and did not significantly differ between the urban and rural cohorts. Instead, specific gravity differed between urban and rural girls, indicating an underlying variability in fluid consumption, individual health, and/or lifestyle factors within the two cohorts.

It is possible that these data represent cohort-specific xenoestrogen exposure in Egypt, as discrepancies in xenoestrogen exposures between urban and rural populations have previously been observed in several studies [42,43]. Lower BPA levels were found in sediments in rural compared to urban Okinawa, Japan, suggesting higher BPA exposure from industrial wastes and less so from agricultural activities [44]. Fu and colleagues traced BPA in atmospheric aerosols from urban, rural, marine, and polar regions around the world, and observed higher atmospheric BPA concentrations in urban regions especially in countries like India where open burning of plastic wastes are common practice [45]. Additionally, the preference of urban populations near Alexandria, Egypt for diets high in meat and fat may expose residents to higher levels of synthetic estrogens reported in poultry and meat [46].

These Egyptian girls, as with other populations around the world, are likely to have exposure to a complex mixture of xenobiotics. When we characterized exposure to phthalates within the same population, urban girls exhibited higher specific gravity adjusted urinary concentrations of several phthalate metabolites compared to rural residents, although these differences were not observed in unadjusted concentrations [47]. Characterization of multiple chemical exposures is necessary to understand the impact of the environment on disease, given that a combination of several low level exposures may contribute to a larger xenobiotic body burden and subsequently influence disease incidence [48,49].

As occupational exposures are unlikely in girls of this age group, the most likely route of exposure is dietary ingestion of BPA [9-11,50]. Several studies show migration of BPA from PVC films in food packaging and cans into foodstuff especially at high temperatures [8,51-53]. Epoxy resin containing BPA is widely used to coat metal products such as canned foods and bottle tops [54]. In addition, some food and drink containers are composed of polycarbonate plastics [9,10]. Therefore, in our study we asked participants about food storage behaviors, and analyzed canned food consumption and food storage in plastic containers as predictors of urinary BPA concentrations. We found a significantly positive association between food storage in plastic containers and BPA concentrations after adjusting for urinary specific gravity, age, BMI, and residential status. A parallel study within this population noted that food storage in plastic was significantly associated with higher urinary phthalate metabolite concentrations [47], suggesting the use of both BPA-containing polycarbonate plastics as well as phthalate-containing PVC storage products by these Egyptian girls. Studies often report BPA exposure from canned food consumption to be relevant [55]; however, our results do not indicate a direct significantly positive association between canned food use and urinary BPA. Interestingly, canned food use improved the association between urinary BPA concentrations and food storage in plastics, acting as a potential confounder. Irrespective of these food consumption and storage behavior associations, BPA concentrations within this Egyptian cohort were significantly lower compared to age- and sex-matched US NHANES data. Comparatively low urinary concentrations of BPA among Egyptian girls may be attributed to the limited use of BPA containing products like thermal paper, and medical equipment, as well as relatively low frequency of plastic food storage and canned food consumption.

Although spot urine samples reflect recent BPA exposure, multiple spot urine testing is recommended for future studies in order to account for temporal variation in exposure [31,56,57]. Besides the small sample size and the collection of only one spot urine sample from each girl, a major limitation of this pilot study is the inclusion of only general behavioral questions. Instead, specific analyses of behavioral and dietary habits are required such as whether food stored in cans and plastics were heated or how many canned food or drink products were consumed. Characterization of the types of food stored and the frequency of use may also be necessary, given that Egyptian adults and children are exposed to different diets especially in rural Egypt [58] and that certain foods are more likely to leach BPA from containers [59].

To the best of our knowledge, this study is one of the first to assess food storage practices and urinary BPA concentrations in girls from a developing country. Many exposure assessment studies focus on developed countries; for example, a previous study found a median unadjusted BPA concentration of 1.8 ng/mL among 1,200 6-8 year old girls from three large US cities [60]. Exploring environmental exposures such as BPA at vulnerable stages of development, including the peripubertal period, will be important for assessing potential risks to disease later in life in developing countries. Reports on the Egyptian food market indicate an increased demand for packaged and convenience foods, and the canned food industry is expected to grow rapidly with the reduction of fresh produce [61]. According to the National Food Consumption Survey, an increasing number of Egyptian households reported consumption of ready-made foods and carbonated soft drinks, and substantial differences in food consumption patterns were observed between urban and rural households over several decades [62]. Given the expected expansion of the Egyptian economy, communities must be cognizant of the potential increase in various environmental exposures and consider appropriate actions to prevent potential adverse health outcomes in the future.

## Conclusions

In this assessment of BPA in girls in the province of Gharbiah, Egypt, similar concentrations of total urinary BPA concentrations were found between girls residing in urban and rural Egypt. The significant association between food storage in plastics and BPA concentrations suggests that diet is an important route for BPA exposure in this population. A closer look at changing food patterns and the consumer market may provide a better understanding of BPA exposure in developing countries.

## Abbreviations

BMI: Body Mass Index; BPA: Bisphenol A; CAPMAS: Central Agency for Public Mobilization and Statistics; CDC: Centers for Disease Control and Prevention; ER: Estrogen Receptor; GM: Geometric Mean; LOD: Limit of Detection; NCEH: National Center for Environmental Health; NHANES: National Health and Nutritional Examination Survey; PVC: Polyvinyl Chloride; SG: Specific Gravity.

## Competing interests

The authors declare that they have no competing interests.

## Authors' contributions

AH, ASS, DCD, IAS, and LSR conceived of this project and designed the study protocols. AH, ASS, and IAS supervised and conducted field collection, including subject recruitment. AMC was responsible for laboratory analysis of BPA. MSN and JAC performed statistical analysis. MSN, KB, DCD, and LSR drafted the manuscript, and all authors edited and approved the final draft of the manuscript.

## Disclaimer

The findings and conclusions in this report are those of the authors and do not necessarily represent the views of the Centers for Disease Control and Prevention.

## References

[B1] Vandenberg LN Chahoud I Heindel JJ Padmanabhan V Paumgartten FJ Schoenfelder G Urinary, circulating, and tissue biomonitoring studies indicate widespread exposure to bisphenol A Environ Health Perspect 2010 118 1055 1070 10.1289/ehp.0901716 20338858 PMC2920080

[B2] Ben-Jonathan N Steinmetz R Xenoestrogens: the emerging story of bisphenol a Trends Endocrinol Metab 1998 9 124 128 10.1016/S1043-2760(98)00029-0 18406253

[B3] Calafat AM Ye X Wong LY Reidy JA Needham LL Exposure of the U.S. population to bisphenol A and 4-tertiary-octylphenol: 2003-2004 Environ Health Perspect 2008 116 39 44 18197297 10.1289/ehp.10753 PMC2199288

[B4] Mountfort KA Kelly J Jickells SM Castle L Investigations into the potential degradation of polycarbonate baby bottles during sterilization with consequent release of bisphenol A Food Addit Contam 1997 14 737 740 10.1080/02652039709374584 9373536

[B5] Carwile JL Luu HT Bassett LS Driscoll DA Yuan C Chang JY Ye X Calafat AM Michels KB Polycarbonate bottle use and urinary bisphenol A concentrations Environ Health Perspect 2009 117 1368 1372 19750099 10.1289/ehp.0900604 PMC2737011

[B6] Sajiki J Yonekubo J Leaching of bisphenol A (BPA) from polycarbonate plastic to water containing amino acids and its degradation by radical oxygen species Chemosphere 2004 55 861 867 10.1016/j.chemosphere.2003.11.065 15041290

[B7] Nerin C Fernandez C Domeno C Salafranca J Determination of potential migrants in polycarbonate containers used for microwave ovens by high-performance liquid chromatography with ultraviolet and fluorescence detection J Agric Food Chem 2003 51 5647 5653 10.1021/jf034330p 12952414

[B8] Lopez-Cervantes J Paseiro-Losada P Determination of bisphenol A in, and its migration from, PVC stretch film used for food packaging Food Addit Contam 2003 20 596 606 10.1080/0265203031000109495 12881134

[B9] Kang JH Kondo F Katayama Y Human exposure to bisphenol A Toxicology 2006 226 79 89 10.1016/j.tox.2006.06.009 16860916

[B10] Vandenberg LN Hauser R Marcus M Olea N Welshons WV Human exposure to bisphenol A (BPA) Reprod Toxicol 2007 24 139 177 10.1016/j.reprotox.2007.07.010 17825522

[B11] Safety F Ottawa Joint FAO/WHO Expert Meeting to Review Toxicological and Health Aspects of Bisphenol A 2010 48

[B12] Biedermann S Tschudin P Grob K Transfer of bisphenol A from thermal printer paper to the skin Anal Bioanal Chem 2010 398 571 576 10.1007/s00216-010-3936-9 20623271

[B13] Calafat AM Weuve J Ye X Jia LT Hu H Ringer S Huttner K Hauser R Exposure to bisphenol A and other phenols in neonatal intensive care unit premature infants Environ Health Perspect 2009 117 639 644 19440505 10.1289/ehp.0800265 PMC2679610

[B14] Fleisch AF Sheffield PE Chinn C Edelstein BL Landrigan PJ Bisphenol A and related compounds in dental materials Pediatrics 2010 126 760 768 10.1542/peds.2009-2693 20819896 PMC4139922

[B15] Vandentorren S Zeman F Morin L Sarter H Bidondo ML Oleko A Leridon H Bisphenol-A and phthalates contamination of urine samples by catheters in the Elfe pilot study: implications for large-scale biomonitoring studies Environ Res 2011 111 761 764 10.1016/j.envres.2011.05.018 21684541

[B16] Welshons WV Thayer KA Judy BM Taylor JA Curran EM vom Saal FS Large effects from small exposures. I. Mechanisms for endocrine-disrupting chemicals with estrogenic activity Environ Health Perspect 2003 111 994 1006 10.1289/ehp.5494 12826473 PMC1241550

[B17] Thomas P Dong J Binding and activation of the seven-transmembrane estrogen receptor GPR30 by environmental estrogens: a potential novel mechanism of endocrine disruption J Steroid Biochem Mol Biol 2006 102 175 179 10.1016/j.jsbmb.2006.09.017 17088055

[B18] Matsushima A Kakuta Y Teramoto T Koshiba T Liu X Okada H Tokunaga T Kawabata S Kimura M Shimohigashi Y Structural evidence for endocrine disruptor bisphenol A binding to human nuclear receptor ERR gamma J Biochem 2007 142 517 524 10.1093/jb/mvm158 17761695

[B19] Richter CA Birnbaum LS Farabollini F Newbold RR Rubin BS Talsness CE Vandenbergh JG Walser-Kuntz DR vom Saal FS In vivo effects of bisphenol A in laboratory rodent studies Reprod Toxicol 2007 24 199 224 10.1016/j.reprotox.2007.06.004 17683900 PMC2151845

[B20] Wetherill YB Akingbemi BT Kanno J McLachlan JA Nadal A Sonnenschein C Watson CS Zoeller RT Belcher SM In vitro molecular mechanisms of bisphenol A action Reprod Toxicol 2007 24 178 198 10.1016/j.reprotox.2007.05.010 17628395

[B21] Brody JG Moysich KB Humblet O Attfield KR Beehler GP Rudel RA Environmental pollutants and breast cancer: epidemiologic studies Cancer 2007 109 2667 2711 10.1002/cncr.22655 17503436

[B22] Dey S Zhang Z Hablas A Seifeldein IA Ramadan M El-Hamzawy H Soliman AS Geographic patterns of cancer in the population-based registry of Egypt: Possible links to environmental exposures Cancer Epidemiol 2011 35 254 264 10.1016/j.canep.2010.09.010 21036119

[B23] Lang IA Galloway TS Scarlett A Henley WE Depledge M Wallace RB Melzer D Association of urinary bisphenol A concentration with medical disorders and laboratory abnormalities in adults JAMA 2008 300 1303 1310 10.1001/jama.300.11.1303 18799442

[B24] Braun JM Kalkbrenner AE Calafat AM Yolton K Ye X Dietrich KN Lanphear BP Impact of early-life bisphenol a exposure on behavior and executive function in children Pediatrics 2011 128 873 882 10.1542/peds.2011-1335 22025598 PMC3208956

[B25] Meeker JD Yang T Ye X Calafat AM Hauser R Urinary concentrations of parabens and serum hormone levels, semen quality parameters, and sperm DNA damage Environ Health Perspect 2011 119 252 257 20876036 10.1289/ehp.1002238 PMC3040614

[B26] Diamanti-Kandarakis E Bourguignon JP Giudice LC Hauser R Prins GS Soto AM Zoeller RT Gore AC Endocrine-disrupting chemicals: an Endocrine Society scientific statement Endocr Rev 2009 30 293 342 10.1210/er.2009-0002 19502515 PMC2726844

[B27] Dey S Soliman AS Merajver SD Xenoestrogens may be the cause of high and increasing rates of hormone receptor positive breast cancer in the world Med Hypotheses 2009 72 652 656 10.1016/j.mehy.2008.10.025 19155145

[B28] Dey S Zhang Z Hablas A Seifeldein IA Ramadan M El-Hamzawy H Soliman AS Geographic patterns of cancer in the population-based registry of Egypt: Possible links to environmental exposures Cancer Epidemiol 2011 35 254 264 10.1016/j.canep.2010.09.010 21036119

[B29] Dey S Soliman AS Hablas A Seifeldein IA Ismail K Ramadan M El-Hamzawy H Wilson ML Banerjee M Boffetta P Harford J Merajver SD Urban-rural differences in breast cancer incidence in Egypt (1999-2006) Breast 2010 19 417 423 10.1016/j.breast.2010.04.005 20452771 PMC3209663

[B30] Ye X Kuklenyik Z Needham LL Calafat AM Automated on-line column-switching HPLC-MS/MS method with peak focusing for the determination of nine environmental phenols in urine Anal Chem 2005 77 5407 5413 10.1021/ac050390d 16097788

[B31] Mahalingaiah S Meeker JD Pearson KR Calafat AM Ye X Petrozza J Hauser R Temporal variability and predictors of urinary bisphenol A concentrations in men and women Environ Health Perspect 2008 116 173 178 18288314 10.1289/ehp.10605 PMC2235217

[B32] Miller RC Brindle E Holman DJ Shofer J Klein NA Soules MR O'Connor KA Comparison of specific gravity and creatinine for normalizing urinary reproductive hormone concentrations Clin Chem 2004 50 924 932 10.1373/clinchem.2004.032292 15105350

[B33] Ouchi K Watanabe S Measurement of bisphenol A in human urine using liquid chromatography with multi-channel coulometric electrochemical detection J Chromatogr B Analyt Technol Biomed Life Sci 2002 780 365 370 10.1016/S1570-0232(02)00547-0 12401363

[B34] Cantonwine D Meeker JD Hu H Sanchez BN Lamadrid-Figueroa H Mercado-Garcia A Fortenberry GZ Calafat AM Tellez-Rojo MM Bisphenol a exposure in Mexico City and risk of prematurity: a pilot nested case control study Environ Health 2010 9 62 10.1186/1476-069X-9-62 20955576 PMC2965706

[B35] Ye X Pierik FH Hauser R Duty S Angerer J Park MM Burdorf A Hofman A Jaddoe VW Mackenbach JP Steegers EA Tiemeier H Longnecker MP Urinary metabolite concentrations of organophosphorous pesticides, bisphenol A, and phthalates among pregnant women in Rotterdam, the Netherlands: the Generation R study Environ Res 2008 108 260 267 10.1016/j.envres.2008.07.014 18774129 PMC2628162

[B36] Yang M Kim SY Lee SM Chang SS Kawamoto T Jang JY Ahn YO Biological monitoring of bisphenol a in a Korean population Arch Environ Contam Toxicol 2003 44 546 551 10.1007/s00244-002-2124-0 12712285

[B37] Volkel W Bittner N Dekant W Quantitation of bisphenol A and bisphenol A glucuronide in biological samples by high performance liquid chromatography-tandem mass spectrometry Drug Metab Dispos 2005 33 1748 1757 10.1124/dmd.105.005454 16103135

[B38] Ibarluzea Jm J Fernandez MF Santa-Marina L Olea-Serrano MF Rivas AM Aurrekoetxea JJ Exposito J Lorenzo M Torne P Villalobos M Pedraza V Sasco AJ Olea N Breast cancer risk and the combined effect of environmental estrogens Cancer Causes Control 2004 15 591 600 15280638 10.1023/B:CACO.0000036167.51236.86

[B39] Fernandez SV Russo J Estrogen and xenoestrogens in breast cancer Toxicol Pathol 2010 38 110 122 10.1177/0192623309354108 19933552 PMC2907875

[B40] Tokunaga M Land CE Tokuoka S Nishimori I Soda M Akiba S Incidence of female breast cancer among atomic bomb survivors, 1950-1985 Radiat Res 1994 138 209 223 10.2307/3578591 8183991

[B41] Cohn BA Wolff MS Cirillo PM Sholtz RI DDT and breast cancer in young women: new data on the significance of age at exposure Environ Health Perspect 2007 115 1406 1414 17938728 10.1289/ehp.10260 PMC2022666

[B42] Rozati R Reddy PP Reddanna P Mujtaba R Role of environmental estrogens in the deterioration of male factor fertility Fertil Steril 2002 78 1187 1194 10.1016/S0015-0282(02)04389-3 12477510

[B43] Cruz S Lino C Silveira MI Evaluation of organochlorine pesticide residues in human serum from an urban and two rural populations in Portugal Sci Total Environ 2003 317 23 35 10.1016/S0048-9697(03)00334-6 14630410

[B44] Kitada Y Kawahata H Suzuki A Oomori T Distribution of pesticides and bisphenol A in sediments collected from rivers adjacent to coral reefs Chemosphere 2008 71 2082 2090 10.1016/j.chemosphere.2008.01.025 18325564

[B45] Fu P Kawamura K Ubiquity of bisphenol A in the atmosphere Environ Pollut 2010 158 3138 3143 10.1016/j.envpol.2010.06.040 20678833

[B46] Sadek IA Survey of hormonal levels in meat and poultry sold in Alexandria. Egypt East Mediterr Health J 1998 4 239

[B47] Colacino JA Soliman AS Calafat AM Nahar MS Van Zomeren-Dohm A Hablas A Seifeldin IA Rozek LS Dolinoy DC Exposure to phthalates among premenstrual girls from rural and urban Gharbiah. Egypt: A pilot exposure assessment study Environ Health 2011 10 40 10.1186/1476-069X-10-40 21575223 PMC3112381

[B48] Darbre PD Charles AK Environmental oestrogens and breast cancer: evidence for combined involvement of dietary, household and cosmetic xenoestrogens Anticancer Res 2010 30 815 827 20393002

[B49] Kortenkamp A Breast cancer, oestrogens and environmental pollutants: a re-evaluation from a mixture perspective Int J Androl 2006 29 193 198 10.1111/j.1365-2605.2005.00613.x 16466540

[B50] Morgan MK Jones PA Calafat AM Ye X Croghan CW Chuang JC Wilson NK Clifton MS Figueroa Z Sheldon LS Assessing the Quantitative Relationships between Preschool Children's Exposures to Bisphenol A by Route and Urinary Biomonitoring Environ Sci Technol 2011 45 5309 5316 10.1021/es200537u 21612268

[B51] Munguia-Lopez EM Gerardo-Lugo S Peralta E Bolumen S Soto-Valdez H Migration of bisphenol A (BPA) from can coatings into a fatty-food simulant and tuna fish Food Addit Contam 2005 22 892 898 10.1080/02652030500163674 16192075

[B52] Munguia-Lopez EM Soto-Valdez H Effect of heat processing and storage time on migration of bisphenol A (BPA) and bisphenol A-diglycidyl ether (BADGE) to aqueous food simulant from Mexican can coatings J Agric Food Chem 2001 49 3666 3671 10.1021/jf0009044 11513645

[B53] Guart A Bono-Blay F Borrell A Lacorte S Migration of plasticizersphthalates, bisphenol A and alkylphenols from plastic containers and evaluation of risk Food Addit Contam Part A Chem Anal Control Expo Risk Assess 2011 28 676 685 21400322 10.1080/19440049.2011.555845

[B54] Kang JH Kito K Kondo F Factors influencing the migration of bisphenol A from cans J Food Prot 2003 66 1444 1447 12929833 10.4315/0362-028x-66.8.1444

[B55] Carwile JL Ye X Zhou X Calafat AM Michels KB Canned soup consumption and urinary bisphenol A: a randomized crossover trial JAMA 2011 306 2218 2220 22110104 10.1001/jama.2011.1721 PMC3367259

[B56] Teitelbaum SL Britton JA Calafat AM Ye X Silva MJ Reidy JA Galvez MP Brenner BL Wolff MS Temporal variability in urinary concentrations of phthalate metabolites, phytoestrogens and phenols among minority children in the United States Environ Res 2008 106 257 269 10.1016/j.envres.2007.09.010 17976571

[B57] Ye X Wong LY Bishop AM Calafat AM Variability of urinary concentrations of bisphenol A in spot samples, first morning voids, and 24-hour collections Environ Health Perspect 2011 119 983 988 10.1289/ehp.1002701 21406337 PMC3223007

[B58] Sukkary-Stolba S Food classifications and the diets of young children in rural Egypt Soc Sci Med 1987 25 401 404 10.1016/0277-9536(87)90278-4 3686088

[B59] Noonan GO Ackerman LK Begley TH Concentration of bisphenol A in highly consumed canned foods on the U.S. market J Agric Food Chem 2011 59 7178 7185 10.1021/jf201076f 21598963

[B60] Wolff MS Teitelbaum SL Pinney SM Windham G Liao L Biro F Kushi LH Erdmann C Hiatt RA Rybak ME Calafat AM Investigation of relationships between urinary biomarkers of phytoestrogens, phthalates, and phenols and pubertal stages in girls Environ Health Perspect 2010 118 1039 1046 10.1289/ehp.0901690 20308033 PMC2920905

[B61] Abdel-Aziz H Egypt: An overview of the food market 2008 Service USC. Alexandria, Egypt: U.S Commercial Service in Alexandria, Egypt. 3

[B62] Galal OM The nutrition transition in Egypt: obesity, undernutrition and the food consumption context Public Health Nutr 2002 5 141 148 12027277 10.1079/PHN2001286

